# Low Dose Interleukin-2 Ameliorates Sjögren’s Syndrome in a Murine Model

**DOI:** 10.3389/fmed.2022.887354

**Published:** 2022-05-19

**Authors:** Yifan Wang, Ruiling Feng, Gong Cheng, Bo Huang, Jiayi Tian, Yuzhou Gan, Yuebo Jin, Miao Miao, Xia Zhang, Xiaolin Sun, Jing He, Zhanguo Li

**Affiliations:** Department of Rheumatology & Immunology, Peking University People’s Hospital, Beijing, China

**Keywords:** Sjögren’s syndrome, interleukin-2, NOD mice, Treg cell, Tfh cells

## Abstract

Sjögren’s syndrome (SS) is a systemic autoimmune disease with no efficient treatment, and it is associated with dysregulated immune cells and impaired interleukin (IL)-2 signaling. IL-2 is critical for the development and maintenance of Treg cells. The use of low dose of IL-2 (LDIL-2) in the treatment of autoimmune diseases is promising, but the efficacy and mechanism in SS therapy are still to be confirmed. This study aims to investigate the therapeutic effect of LDIL-2 on SS in NOD (non-obese diabetic) mice. NOD mice (female, 8 weeks old) were randomly assigned into three groups (*n* = 8). Low dose of IL-2 (LDIL-2), high dose of IL-2 (HDIL-2), and isometric sterile water (control) were administered subcutaneously daily from week 8 to week 16. LDIL-2 administration significantly recovered the reduction in saliva flow and suppressed lymphocyte inflammation of the submandibular glands (SMGs) when compared with those treated with sterile water as controls (*p* < 0.05). SS related biomarkers including ANA, Anti-SSA/Ro, and Anti-SSB/La also declined (*p* < 0.05). In the low dose of IL-2 treated group, the proportion of CD4+CD25+Foxp3+Tregs in both spleen and cervical-lymph-node were higher than control mice (*p* < 0.05). Furthermore, CD4+Bcl-6+PD-1+CXCR5+Tfh cells, CD4+IFN-γ+Th1 cells, and CD4+IL-17A+Th17 cells were significantly reduced in LDIL-2 group (*p* < 0.05). Analysis of the SMGs biopsies showed significantly decreased inflammation scores after LDIL-2 administration and an increase of Tregs with immunohistochemical staining. Our findings provide *in vivo* evidence that LDIL-2 was an effective therapeutic intervention for SS observed in NOD mice and may restore immune balance through the promotion of Treg and suppression of germinal center (GC) B cells and effector T cells.

## Introduction

Sjögren’s syndrome (SS) is a chronic, systemic autoimmune disease characterized by loss of exocrine gland function. T and B lymphocytes are primarily responsible for infiltrating exocrine glands at different stages of inflammation in SS patients. Patients with SS may also show a diversity of extra-glandular manifestations, such as lung disease, kidney disease, arthralgia, and fatigue (1, 2).

As a hallmark of SS, B-cell hyperactivity causes hypergammaglobulinemia (3, 4), autoantibody production, increased serum interleukin-6 (IL-6), IL-17, IL-21, IFN-α levels, and an increased risk of lymphoma, particularly B-cell-derived non-Hodgkin lymphomas (5, 6). Mechanistically, activated T cells providing stimulation to B cells are central to these abnormalities (7). In patients with SS, we and others have described that IL-17 is increased in the circulation, and is correlated with B cell activation and autoantibodies production (8, 9). In addition, inflammatory lesions of the salivary glands in SS patients showed increased levels of IL-17 expression (9, 10). Other studies described follicular helper T cells (Tfh) in the circulation of SS patients and correlated their presence with higher titers of autoantibodies, inflammatory cytokines, and more severe disease. In addition to hyperactivity of effector subsets, impaired numbers and functions of regulatory T (Treg) cells have been noted in SS patients (11–13). Promotion of Tregs in patients with SS is considered one potential approach to reduce T and B cell hyper-reactivity in SS.

NOD (Non-obese diabetic mice, NOD/ShiLtJ mice) are characterized by profound secretory gland dysfunction associated with lymphocyte infiltration, which resembles the symptoms of SS (14, 15). In addition, many SS-related autoantibodies, such as antinuclear antibodies (ANA), anti-SSA/Ro, anti-SSB/La, are present in the serum of NOD mice (16, 17). As mouse models of SS, NOD mice have been extensively investigated, and many factors, including T cells, B cells, various cytokines, and dysregulated homeostasis in exocrine glands, have been shown to contribute to the development of the disease (18–23).

IL-2 is a critical cytokine for the differentiation and maintenance of Foxp3+Treg cells (24–26). Decreased intra-islet Tregs function in NOD mice could be corrected with IL-2 administration and the severity of diabetes reduced in mice (27). In human clinical studies, low dose of IL-2 (LDIL-2) has been used in small cohorts of patients with systemic lupus erythematosus (SLE), chronic graft versus host disease (GVHD), Type 1 diabetes (T1D), and Hepatitis C virus (HCV) related vasculitis. These studies demonstrated that LDIL-2 increased the number of Tregs and improved disease manifestations in all these autoimmune disorders (28–31). Recently, we observed that administration of low dose of IL-2 could also inhibit Tfh and Th17 cell differentiation in SLE (31). However, therapies available for the treatment of SS are currently inadequate. We hypothesized that low dose of IL-2 might reduce both the aberrant T and B cells’ responses in SS. Based on the findings from those studies, here, we use NOD mice to investigate the therapeutic effect of low dose of IL-2 on SS.

## Materials and Methods

### Mice and Experimental Protocol

NOD mice (female, NOD/ShiLtJ mice, 8 weeks old, weight 18–22 g, No. N000235) were obtained from the Model Animal Research Center of Nanjing University (Nanjing, China) and were maintained at the Laboratory Animal Center in Peking University (Beijing, China). Recombinant human interleukin-2 (rhIL-2) was purchased from SL. PHARM (SL PHARM, Beijing, China) and dissolved in sterile water. Mice were in the same genetic background and allocated into each experimental group by randomization (*n* = 8). Low dose of rhIL-2 (30,000 IU/d), high dose of rhIL-2 (300,000 IU/d), and isometric sterile water was administered subcutaneously injection daily from week 8 to week 16 (32–34). IL-2-treated mice were analyzed at age of 16 weeks with blinding on experimental groups. All animal experiments were approved by the Institutional Ethics Committee of Peking University (permit number: 2017PHC062).

### Saliva Measurement

Saliva flow rates were measured every 2 weeks. Saliva secretion was induced by intraperitoneal injection with pilocarpine (Sigma-Aldrich, St. Louis, MO, United States) at a dose of 0.5 mg/kg body weight after anesthetization. Stimulated whole saliva was gravimetrically collected using a 20-μL sized pipet trip from the oral cavity for 15 min at room temperature. The body weight was recorded at the same time and the volume of saliva was normalized to the body weight.

### Histological and Immunohistochemical Analysis

Submandibular glands (SMGs) were surgically removed, fixed in 4% paraformaldehyde, and embedded in paraffin. Mouse SMG tissues were prepared for sectioning with hematoxylin and eosin (H&E) staining. Lymphocytic infiltration areas were captured and assessed under the photomicroscope. The histological grade was determined as follows: 1 = 1–5 leukocytic foci (infiltrated lymphocytes 50 or more per 4 mm^2^) were seen; 2 = more than 5 foci, no significant parenchymal destruction; 3 = multiple confluent foci and moderate degeneration of parenchymal tissue; 4 = extensive lymphocytes infiltration of the gland and parenchymal destruction, as described previously (35–37). For quantitation of inflammation, the proportion of inflammation area was calculated to the total area of the section except for fatty infiltration (38). SMG tissues were subsequently conducted with immunohistochemical staining. The de-paraffinized sections were incubated with anti-CD4+ (1:300 ab#183685, Abcam, Shanghai, China), anti- Foxp3+ antibodies (1:100 ab#22510, Abcam, Shanghai, China) at 4^°^C overnight according to the manufacturer’s instructions. Images were captured at 400× magnification under a photomicroscope (original magnification, ×200). The number of CD4+and Foxp3+ cells and the total number of mononuclear inflammatory cells were counted using Image J software and recorded. Quantification of positively stained areas in the sections was measured with NDP.View2 software (NDP.View2, Hamamatsu, Japan) as described previously (39).

### Flow Cytometry

Murine spleens and cervical lymph nodes (cLN) were dissected freshly and prepared for flow cytometry. Cells were collected and homogenized in RPMI-1640 medium (Gibco, Thermo Fisher Scientific, Waltham, MA, United States) supplemented with 10% fetal bovine serum (FBS, Gibco) and 1% penicillin-streptomycin (Gibco). Splenocytes and cLNs cells were stained with a combination of fluorescence-conjugated monoclonal antibodies to surface markers CD3 (PE-Cy7 ab#1727462), CD4 (BV421 ab#2739780, BV650 ab#2716859), CD8 (APC-Cy7 ab#396769, APC ab#398527), PD-1 (APC ab#2869928), CD25 (PE-Cy7 ab#394509), CXCR5 (BUV395 ab#2738521), CD95 (PE ab#395330), GL-7 (AF488 ab#394981), and B220 (Percp-cy5.5 ab#394457) (BD Biosciences, Franklin Lakes, NJ, United States) at 4^°^C for 30 mins. Intracellular and nuclear staining was subsequently conducted using monoclonal antibodies against Foxp3 (PE ab#11151905), IFN-γ (BV711 ab#2738752), and IL-17A (BV421 ab#2687547) (BD Biosciences, Franklin Lakes, NJ, United States) as previously described (40). Flow cytometry gating strategy is available in Supplementary Figures 1–3. Stained cells were acquired and analyzed using a CytoFLEX flow cytometer (Beckman Coulter, IN, United States) and Kaluza Analysis software (Beckman Coulter, Brea, CA, United States).

### Autoantibodies Quantification

The mice sera were diluted at 1:40 and prepared subsequently for immunofluorescence. The concentrations of ANA were measured by immunofluorescence staining with human HEp-2 human epithelial cell-substrate slides as described previously (41, 42). The concentrations were diluted as 1:10, 1:32, 1:100, 1: 320, and 1:1,000. 1:100 and higher was regarded as positive. The results of slides were evaluated at 400× magnification by professional inspectors under blinding. The serum levels of autoantibodies against SSA/Ro (total IgG) and anti-SSB/La (total IgG) was detected by a commercially available ELISA kit (Euroimmun, Lubeck, Germany) according to the manufacturer’s instructions as described previously (43–45).

### Statistical Analysis

All the data were expressed as the mean ± standard deviation (SD) and were analyzed using the GraphPad Prism 8.0 software (GraphPad Software Inc., San Diego, CA, United States) or IBM SPSS Statistics 23.0 software (SPSS Inc., Armonk, NY, United States). Statistical comparisons were performed with the student’s *t*-test, the non-parametric Mann-Whitney *U* test, and the Kruskal-Wallis test. Data correlation analysis with non-parameter Spearman correlation coefficient. Both tests were two-tailed at 95% confidence interval, and *p* ≤ 0.05 (*p < 0.05, **p < 0.01, and ***p < 0.001) considered to be statistically significant.

## Results

### Low Dose IL-2 Sustained Salivary Flow Rate and Decreased Salivary Gland Inflammation in Non-obese Diabetic Mice

**FIGURE 1:**
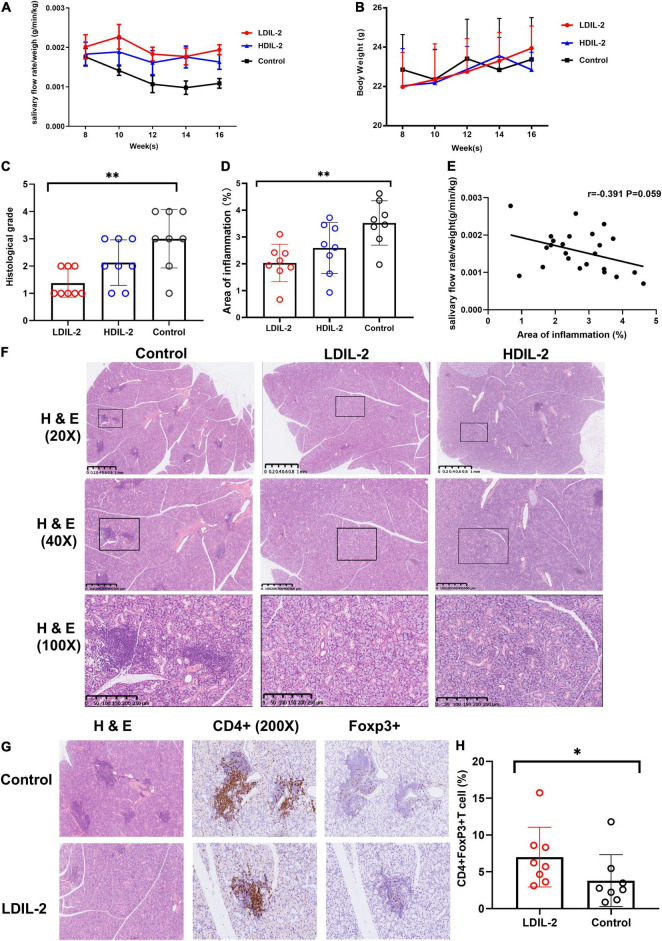
Low dose of IL-2 sustains salivary flow rate and improves salivary gland inflammation in NOD mice. Eight-week-old mice were subcutaneously administered LDIL-2 (30,000 IU/d), HDIL-2 (300,000 IU/d) and isometric sterile water daily for 8 weeks. **(A)** The salivary flow rate normalized to body weight was collected every 2 weeks after pilocarpine stimulation (*n* = 8 per group). **(B)** The body weight was measured every 2 weeks and showed a stable increase after LDIL-2 administration. **(C,D,F)** Mouse SMGs tissues were stained with hematoxylin and eosin (H&E). Histological analysis of SMGs from LDIL-2 and sterile water-treated mice was conducted and a strikingly lower histological score was shown in LDIL-2 group. The lymphocytic foci were circled and visualized under 20× objective, 40× objective, and 100× objective to evaluate the degree of lymphocytic infiltration. The number of lymphocytic foci and the area of lymphocytic infiltration in response to LDIL-2 were significantly reduced as compared with control group. **(E)** Correlation analysis between the proportion of inflammation area and salivary flow rates. **(G)** Immunohistochemical method was used to evaluate the abundance of Foxp3+Treg cells in the SMGs showed a similar trend of suppressed lymphocytic infiltration under 400× objective. **(H)** Distribution of CD4+Foxp3+cells frequencies in SMGs between LDIL-2 group and control groups. Data were analyzed with non-parametric Mann–Whitney test or Student’s *t*-test, with *p < 0.05 indicating a significant difference. SMGs, submandibular glands; LDIL-2, low dose of interleukin-2; HDIL-2, high dose of interleukin-2 (*p < 0.05, **p < 0.01).

To explore the possible effect of IL-2 in restoring the function of SMG, we measured saliva flow rates every 2 weeks. Results showed that the amounts of saliva collected for 15 min after pilocarpine stimulation witnessed a significant increase in IL-2 treated mice, while the amounts of saliva decreased obviously in sterile water-treated mice (control group) from weeks 10 to 16 (Figure 1A). In addition, there was also a steady increase in body weight following low dose of IL-2 administration in week 14 and week 16, but there were no statistically significant differences (Figure 1B).

We monitored random blood glucose levels in mice every two weeks in our study. Compared to the control group, the mice in the IL-2 treated group had relatively low glucose levels, without statistically significant differences (Supplementary Figure 4). All of the mice in the study did not achieve the diagnosed blood glucose levels for diabetes and probably did not reach the development stage of the disease.

Histological examination of the SMGs was performed at weeks 16 and SMGs tissue was stained with H&E as previously described. Histological score revealed that lymphocytic infiltration was strikingly lower in the low dose of IL-2 treated group (histological score: LDIL-2 1.38 ± 0.52, control 3.01 ± 1.07, *p* = 0.003) compared to the control group. Such pathologic changes were much improved in mice treated with IL-2. The mice receiving high dose of IL-2 had similar histological conditions to those from the control group (histological score: HDIL-2 2.13 ± 0.83, *p* = 0.147; Figure 1C). Quantification of the area of inflammation showed that the percent of inflammation area was significantly lower in the LDIL-2 group (area of inflammation: LDIL-2 2.04 ± 0.70, control 3.46 ± 0.86, *p* = 0.009; Figure 1D). There was no statistical significance between HDIL-2 and control groups (area of inflammation: HDIL-2 2.59 ± 0.95, *p* = 0.152). Interestingly, as the area of inflammation went up, a reverse trend was found in salivary flow rates. The severity of inflammation in submandibular glands correlated with salivary flow rates but this correlation failed to reach statistical significance (*r* = –0.391, *p* = 0.059; Figure 1E). Compared with the control group, SMGs from IL-2 treated mice showed obvious fewer lymphocytic infiltration and foci under a photomicroscope, indicative of suppressed lymphocyte inflammation in SMGs tissue (Figure 1F). These results suggest that LDIL-2 has a protective effect on inflammatory responses of SS.

### Low Dose IL-2 Enhanced Tregs in the Spleen, Cervical Lymph Nodes, and Submandibular Glands

**FIGURE 2:**
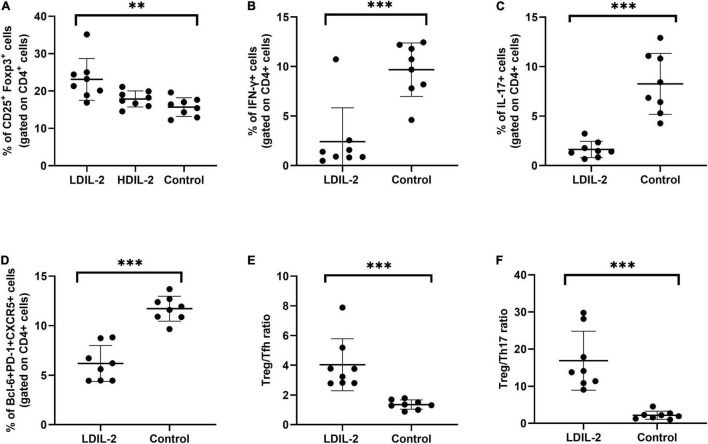
Low dose of IL-2 controls CD4+ T cell differentiation in spleen. Spleens were removed at week 16 and stained with antibodies CD4, CD8, PD-1, and intracellularly stained anti-Foxp3, CXCR5 antibodies. Flow cytometry were performed and revealed the frequency of Treg (CD4+CD25+Foxp3+), Tfh (CD4+Bcl-6+PD-1+CXCR5+), Th1 (CD4+IFN-γ+), Th17 (CD4+IL-17A+), Treg to Tfh ratio, and Treg to Th17 ratio. **(A)** In LDIL-2 treated mice, amount of Tregs was dramatically increased. **(B–D)** Decreased populations of Th1, Th17, and Tfh cell were shown in LDIL-2 group compared with control group. **(E,F)** The ratio of Treg to Tfh cells and Treg to Th17 cells presented a substantial increase in LDIL-2 treated mice. Data were analyzed with non-parametric Mann–Whitney test or Student’s *t*-test with *p < 0.05 indicating a significant difference. LDIL-2, low dose of interleukin-2; HDIL-2, high dose of interleukin-2 (**p < 0.01, and ***p < 0.001).

**FIGURE 3:**
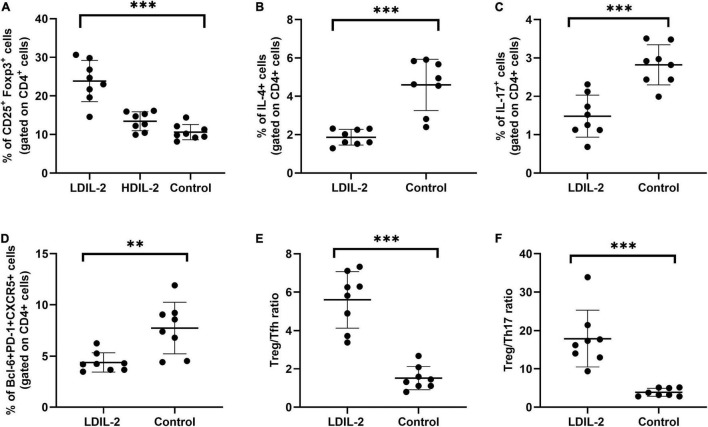
Low dose of IL-2 enhances the percentage of Treg cells and inhibits Th1 and Th17 populations in cLNs. cLN cells were isolated from NOD mice at week 16 and CD4+CD25+Foxp3+Tregs, Tfh (CD4+Bcl-6+PD-1+CXCR5+), Th1 (CD4+IFN-γ+helper T) cells and Th17 (CD4+IL-17A+helper T) cells were subjected to flow cytometry. **(A,D)** LDIL-2 administration increased the prevalence of Tregs and Tfh cells in cLNs from mice compared with control mice. **(B,C)** The populations of Th1 and Th17 cells were suppressed in LDIL-2 treated mice. **(E,F)** LDIL2-treated mice showed a significantly higher ratio of Treg/Tfh and Treg/Th17. Data were analyzed with non-parametric Mann–Whitney test with *p < 0.05 indicating a significant difference. cLNs, cervical lymph nodes; LDIL-2, low dose of interleukin-2 (**p < 0.01, and ***p < 0.001).

To address the impact of IL-2-treatment in Tregs population dynamics, we analyzed the CD4+Foxp3+T cells in SMGs by immunohistochemistry (IHC), and CD4+CD25+Foxp3+ regulatory T (Treg) cells in the spleen and cLNs by flow cytometry. Compared to the control group, the immunohistochemical analysis of images showed that SMGs of low dose of IL-2 treated NOD mice contained considerably more positive CD4+Foxp3+T cells (*p*_LDIL–2 vs. control_ = 0.0281) (Figures 1G,H). Consistent with the increase of Tregs in IHC, LDIL-2 administration also significantly expanded Tregs in spleen and cLNs from NOD mice than did their sterile water counterparts (spleen: *p*_LDIL–2vs. control_ = 0.002; cLNs: *p*_LDIL–2vs. control_ = 0.001; Figures 2A, 3A).

### Low Dose IL-2 Decreased Th1 and Th17 Populations in the Spleen and Cervical Lymph Nodes

To investigate the effects of LDIL-2 on SS development, with the emphasis on regulatory and effector CD4+ T cell subsets, spleen cells, and cLNs cells were evaluated via flow cytometry. Data showed a marked reduction in the frequencies of Th1 (CD4+IFN-γ+T-helper) cells and Th17 (CD4+IL-17A+T-helper) cells in both the spleen and cLNs from LDIL-2 treated mice (spleen: Th1 *p*_LDIL–2vs. control_ = 0.004, Th17 *p*_LDIL–2vs. control_ = 0.001; cLNs: Th1 *p*_LDIL–2vs. control_ = 0.001, Th17 cLNs: *p*_LDIL–2vs. control_ = 0.002; Figures 2B,C, 3B,C). These results indicate that treatment with low dose of IL-2 modulates the differentiation of CD4+ T cell subsets and attenuates infiltration of a specific SS-associated T-cell population *in vivo*.

### Low Dose IL-2 Treatment Reduced Tfh Cells and Restored the Balance Between Tregs and Teff in the Spleen and Cervical Lymph Nodes

**FIGURE 4:**
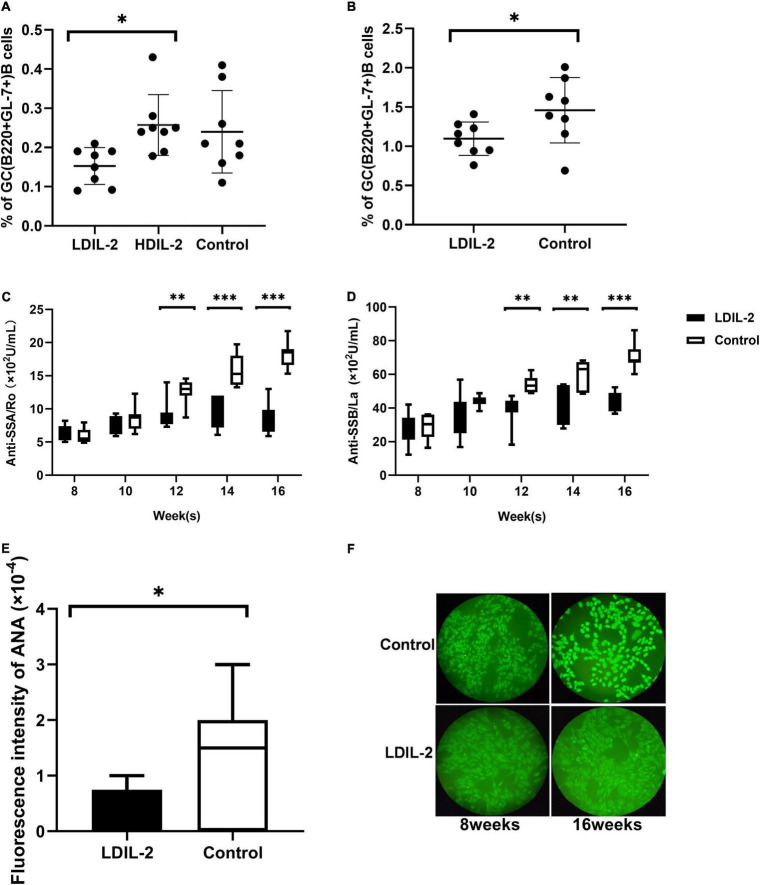
Low dose of IL-2 suppresses GC B Cells population and inhibits SS related autoantibodies production in NOD mice. Splenocytes and cLNs cells obtained from mice euthanized at 16 weeks of age were stained for flow cytometry. **(A)** The analysis indicated that GC B (B220+GL-7+) cells in spleen and cLNs **(B)** were decreased after low-dose IL-2 treatment. **(C,D)** The serum levels of anti-SSA/Ro and anti-SSB/La, determined by ELISA, showed a decreased titer in LDIL-2 treated mice at weeks 16. **(E)** Immunofluorescence assay experienced decreased titer of ANA in LDIL-2 group as compared to control group at weeks 16. The representative anti-ANA images of immunofluorescence staining with human HEp-2 epithelial cells antibodies from each group were shown in **(F)**. Data were analyzed with non-parametric Mann–Whitney test (*n* = 8). *p < 0.05 indicating a significant difference. LDIL-2, low dose of interleukin-2; HDIL-2, high dose of interleukin-2 (*p < 0.05, **p < 0.01, and ***p < 0.001).

Given the potent relation of Tfh on germinal center (GC) B cells, we assessed whether LDIL-2 inhibits the Tfh differentiation in NOD mice. We next analyzed the Tfh (CD4+Bcl-6+PD-1+CXCR5+follicular helper T) cells in low dose of IL-2 treated mice. The frequency of Tfh cells was significantly decreased in both the spleen and cLNs (spleen: *p* = 0.001; cLNs: *p* = 0.003; Figures 2D, 3D), consisted with decreased frequencies of GC B cells (Figures 4A,B). Thus, LDIL-2 treatment may inhibit Tfh and GC responses. Finally, we explored whether LDIL-2 can restore Treg/Teff balance. After treatment with LDIL-2, evidently increased ratios of Tregs to Tfh cells and Tregs to Th17 cells were observed in cLNs and spleen of NOD mice compared to controls (*p* = 0.001, overall; Figures 2E,F, 3E,F).

### Low Dose IL-2 Decreased the Number of Germinal Center B Cells in Non-obese Diabetic Mice and Inhibited the Production of Sjögren’s Syndrome Related Autoantibodies

To clarify whether LDIL-2 could effectively affect the population of B cells, we utilized flow cytometry to analyze its influence on GC B cells. IL-2 administration reduced GC B (B220+GL-7+) cells in in the spleen compared with the control mice (LDIL-2 0.15 ± 0.05, HDIL-2 0.26 ± 0.08, control 0.24 ± 0.11, *p*_LDIL–2vs. control_ = 0.051; Figure 4A). Notably, a similar decrease was observed in the cLNs of the low dose of IL-2 treated mice (LDIL-2 1.10 ± 0.21, control 1.46 ± 0.42, *p* = 0.045; Figure 4B). Furthermore, considering the pivotal role of autoantibody titer during SS development, we measured the concentration of SS-related serum antibodies ANA, anti-SSA/Ro and anti-SSB/La. The titer of anti-SSA/Ro, and anti-SSB/La via ELISA was remarkably lower than those in the control group at weeks 16 (anti-SSA/Ro: *p*_LDIL–2vs. control_ = 0.001; anti-SSB/La: *p*_LDIL–2vs. control_ = 0.001; Figures 4C,D). Accordingly, immunofluorescence analysis demonstrated that ANA in the LDIL-2 group shared a similar tendency at weeks 16 (ANA: *p*_LDIL–2vs. control_ = 0.041; Figures 4E,F). Altogether, these findings underlie the selective suppressive effects of LDIL-2 in the production of serum antibodies during SS development, possibly attributed to impeded GC B cell formation in NOD mice.

## Discussion

Clinical studies of exogenous IL-2 have demonstrated efficacy in patients with SLE, T1D, and GVHD, associated with Tregs expansion. In NOD mice, others have shown that the administration of LDIL-2 promoted Treg cell survival and protected mice from developing diabetes (27). Our study also demonstrated consistent results, with no increase in blood glucose in the LDIL-2 group at 16 week, whereas glucose levels were relatively higher in the controls. It has been demonstrated that low dose of IL-2 can contribute to an increase in Tregs (46). Numerous clinical trials support this concept, and in a phase i/II double-blind, randomized, placebo-controlled trial of low-dose IL-2 in patients with TID, patients were well-tolerated and moderate improvements in rapid C-peptide with IL-2 were observed, along with a significant increase in the proportion of Treg cells (28, 47).

Previous studies showed that Treg cell deficiency leads to various autoimmune and inflammatory diseases. For example, Treg cell-specific deletion on the NOD background mice results in lethal auto-immunity due to defective suppressive abilities of Treg cells in models of diabetes and colitis. In this system, Treg cells progressively lose Foxp3 expression and gain IFN-γ secretion (33). IL-2 is the key cytokine for differentiation, survival, and function of Treg cells (33). Our current studies demonstrated that LDIL-2 can increase the number of Treg cells in NOD mice. The increase in Tregs was also associated with significant improvement in salivary gland secretions and reduction in the lymphocytic infiltration of the salivary glands. In addition, the dosage of LDIL-2 used in these studies also suppressed GC B cells. This suggests that the low dose of IL-2 inhibits B cells differentiation into GC B cells and increases Treg cells in the NOD mouse.

Till now, little is known about the ability of the potent function of low dose of IL-2 to modulate B-cell subsets, which may influence humoral immune abnormalities of SS. In this study, low dose of IL-2 administration reduced the production of SS-related antibodies, including ANA, anti-SSA, and anti-SSB antibodies, which might be a result of GC B cell suppression. Future studies should focus on the impact of LDIL-2 treatment on other immune cell subtypes, such as Tfh, memory B cells, plasma B cells. In the present study, low-dose IL-2 treatment restores circulating Treg cells and the ratio of Treg/Th17 based on a study of 190 pSS patients (48). Similar results were observed in NOD mice that LDIL-2 modulated the differentiation of CD4+ T cells. In light of considerably decreased Tfh cells, increased ratios of Tregs to Tfh cells, and Tregs to Th17 cells in NOD mice compared to controls, we conclude that low-dose IL-2 maintains the balance between Tregs and Teff cells in NOD mice.

The link between circulation Tregs and tissue-resident Tregs is not fully understood in autoimmune diseases. Pathogenic antigens and cytokines may induce the expression of several chemokines favoring massive T cells homing toward damaged tissues, such as the salivary gland (SG) of SS. The characterization of tissue-specific Tregs and their mechanisms of action will have important implications for the maintenance of tissue homeostasis and the resolution of autoimmunity in damaged settings (33). In human studies, Castela et al. showed that LDIL-2 can recruit CD4+CD25+Foxp3+Treg cells into the skin of patients with Alopecia (33). Here, Foxp3 expression was higher in SG of LDIL-2 treated mice than in control, unfortunately, there was no quantitative analysis to compare the differences between the two groups, which suggests that low dose of IL-2 could induce the differentiation of Treg and thereby, displayed substantial improvement of salivary gland function in NOD mice. Thus, low dose of IL-2 can affect abnormal Treg cells universally, both in circulation and in tissues.

In summary, our findings provide *in vivo* evidence that LDIL-2 is effective in SS and may restore immune balance through the promotion of Treg and suppression of GC B cells. This study provides preliminary evidence that LDIL-2 ameliorates inflammation of SS pathology in an experimental animal model. We believe that this novel therapeutic approach has the potential to positively impact clinical application worldwide.

## Data Availability Statement

The raw data supporting the conclusions of this article will be made available by the authors, without undue reservation.

## Ethics Statement

The animal study was reviewed and approved by the Institutional Ethics Committee of Peking University (permit number: 2017PHC062).

## Author Contributions

JH and ZL contributed to the design, analysis, fund support, and conception of the study. YW, RF, and BH contributed to the laboratory work. JT, YG, YJ, MM, and XZ contributed to the data acquisition and performed data analysis. YW, RF, and GC contributed to the manuscript preparation and wrote the manuscript. JH and XS helped and revised the manuscript. All authors contributed to the work and approved the final submitted version of the manuscript.

## Conflict of Interest

The authors declare that the research was conducted in the absence of any commercial or financial relationships that could be construed as a potential conflict of interest.

## Publisher’s Note

All claims expressed in this article are solely those of the authors and do not necessarily represent those of their affiliated organizations, or those of the publisher, the editors and the reviewers. Any product that may be evaluated in this article, or claim that may be made by its manufacturer, is not guaranteed or endorsed by the publisher.
