# Supplementary material for: Low Dose Interleukin-2 Ameliorates Sjögren’s Syndrome in a Murine Model
Source: Front Med (Lausanne). 2022 May 19;9:887354. doi: 10.3389/fmed.2022.887354 (PMC9160330; doi:10.3389/fmed.2022.887354)
Supplement: Supplementary file 1 [file Data_Sheet_1.PDF]

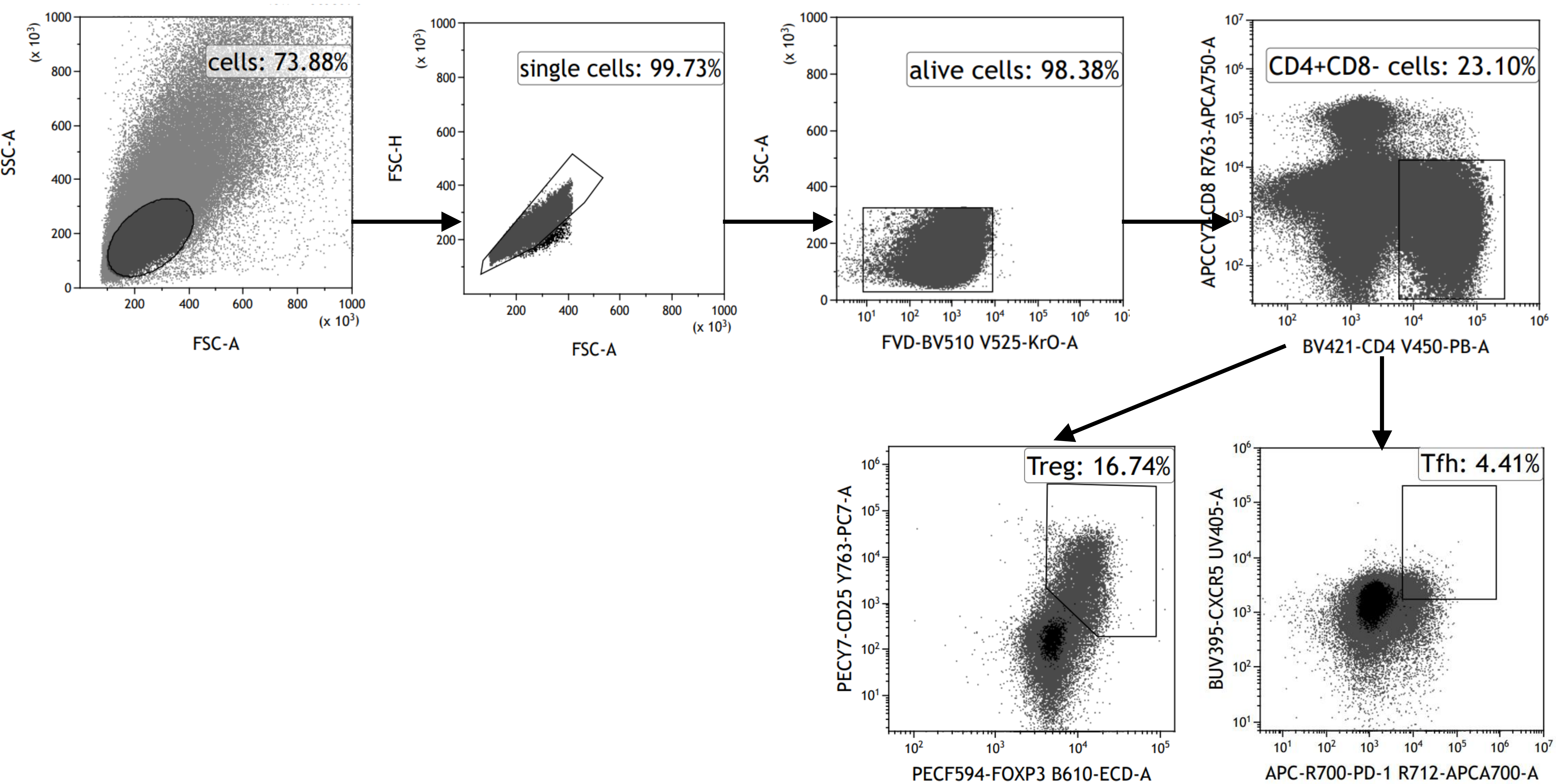

Supplementary Figure 1. Flow cytometry gating strategy for Treg (CD4+CD25+Foxp3+), and Tfh (CD4+Bcl-6+PD-1+CXCR5 +) cells.

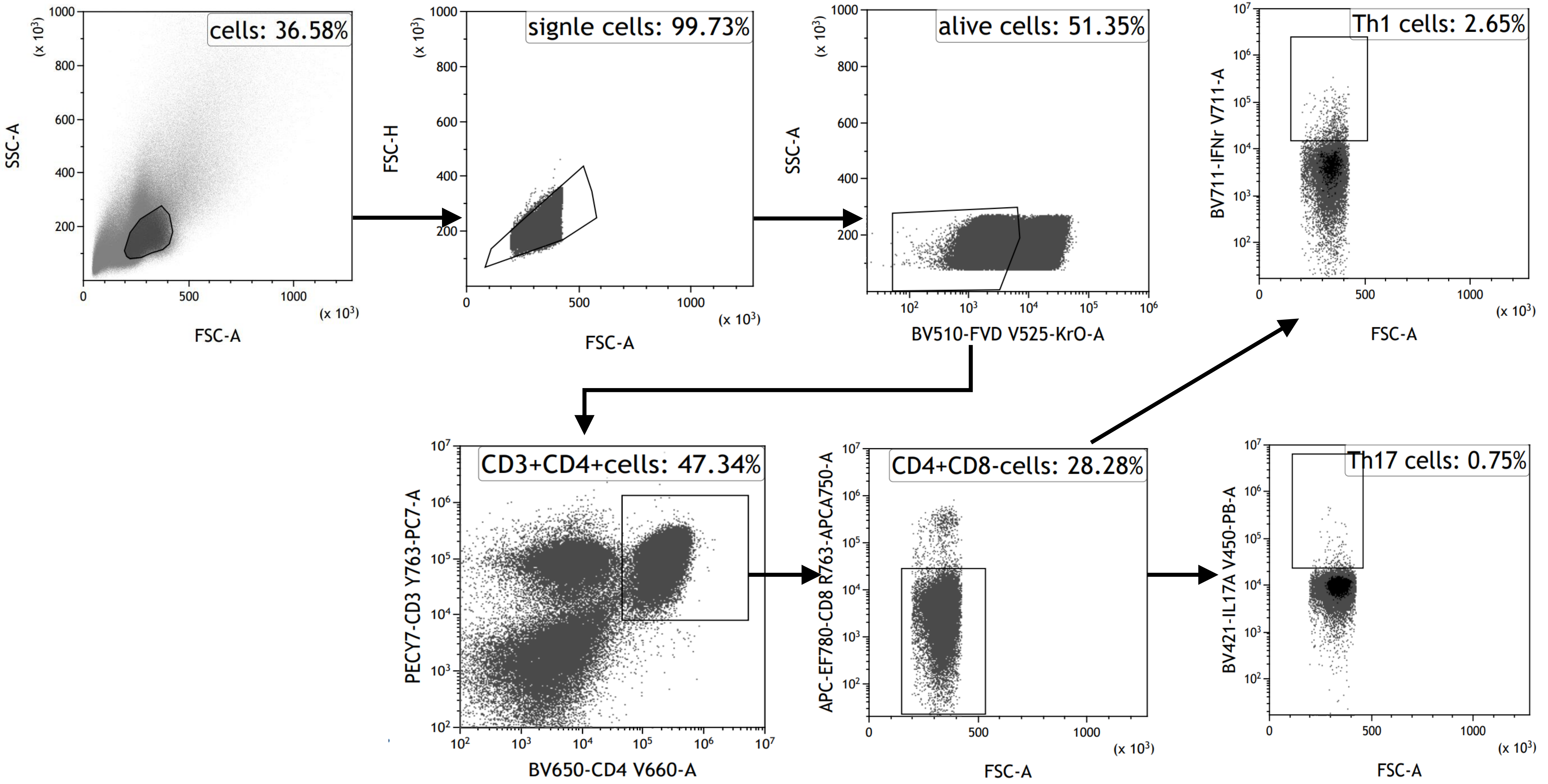

Supplementary Figure 2. Gating strategy for Th1 (CD4+IFN-γ+), and Th17 (CD4+IL-17A+) cells.

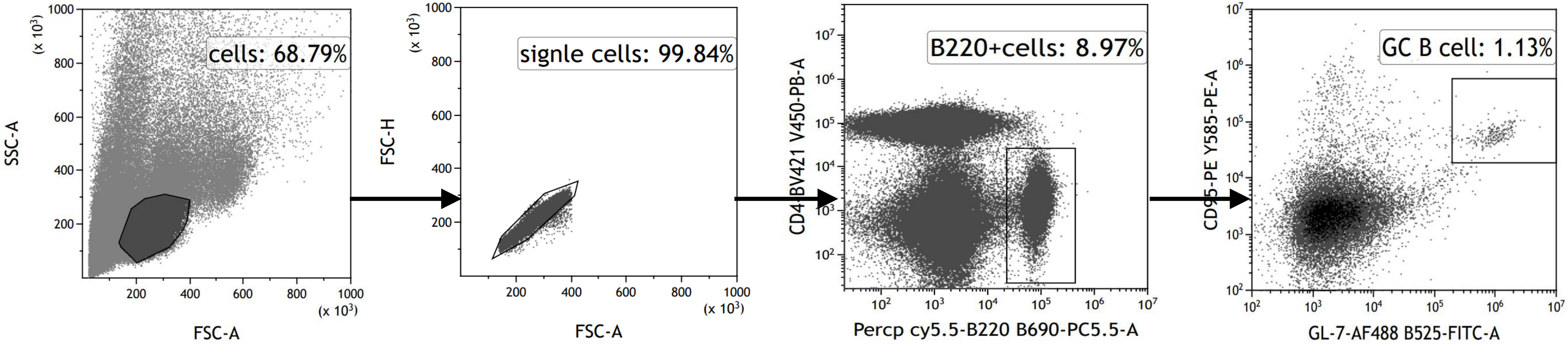

Supplementary Figure 3. Gating strategy for GC B (B220+GL-7+) cells.

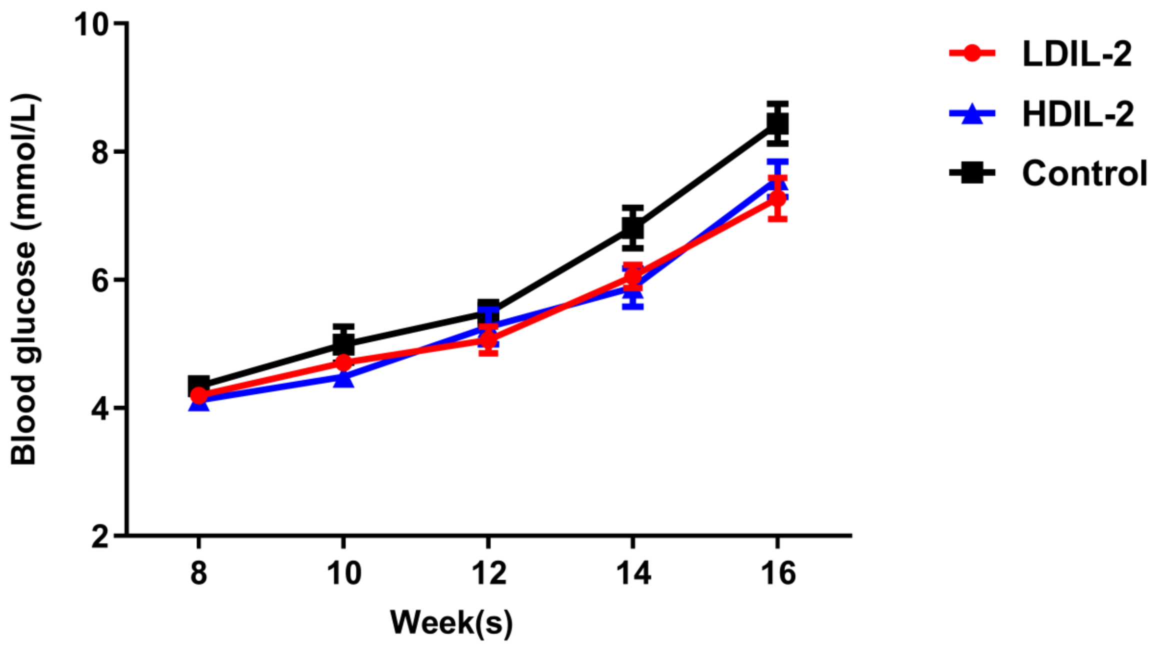

Supplementary Figure 4. Random blood glucose was measured every 2 weeks in NOD mice from 8 weeks to 12 weeks.
